# Enhanced Performance of Reagent-Less Carbon Nanodots Based Enzyme Electrochemical Biosensors

**DOI:** 10.3390/s19245576

**Published:** 2019-12-17

**Authors:** Iria Bravo, Cristina Gutiérrez-Sánchez, Tania García-Mendiola, Mónica Revenga-Parra, Félix Pariente, Encarnación Lorenzo

**Affiliations:** 1 Departamento de Química Analítica y Análisis Instrumental, Universidad Autónoma de Madrid, Cantoblanco, 28049 Madrid, Spain; iria.bravo@uam.es (I.B.); cristina.gutierrezs@uam.es (C.G.-S.); tania.garcia@uam.es (T.G.-M.); monica.revenga@uam.es (M.R.-P.); felix.pariente@uam.es (F.P.); 2 Instituto Madrileño de Estudios Avanzados en Nanociencia (IMDEA-Nanociencia), Faraday, 9, Campus UAM, Cantoblanco, 28049 Madrid, Spain; 3 Institute for Advanced Research in Chemical Sciences (IAdChem), Universidad Autónoma de Madrid, 28049 Madrid, Spain

**Keywords:** carbon nanodots, nanomaterials, oxidoreductase-based biosensors, L-lactate biosensor, electrochemical techniques, lactate oxidase, glucose oxidase, uricase

## Abstract

This work reports on the advantages of using carbon nanodots (CNDs) in the development of reagent-less oxidoreductase-based biosensors. Biosensor responses are based on the detection of H_2_O_2_, generated in the enzymatic reaction, at 0.4 V. A simple and fast method, consisting of direct adsorption of the bioconjugate, formed by mixing lactate oxidase, glucose oxidase, or uricase with CNDs, is employed to develop the nanostructured biosensors. Peripherical amide groups enriched CNDs are prepared from ethyleneglycol bis-(2-aminoethyl ether)-N,N,N′,N′-tetraacetic acid and tris(hydroxymethyl)aminomethane, and used as precursors. The bioconjugate formed between lactate oxidase and CNDs was chosen as a case study to determine the analytical parameters of the resulting L-lactate biosensor. A linear concentration range of 3.0 to 500 µM, a sensitivity of 4.98 × 10^−3^ µA·µM^−1^, and a detection limit of 0.9 µM were obtained for the L-lactate biosensing platform. The reproducibility of the biosensor was found to be 8.6%. The biosensor was applied to the L-lactate quantification in a commercial human serum sample. The standard addition method was employed. L-lactate concentration in the serum extract of 0.9 ± 0.3 mM (n = 3) was calculated. The result agrees well with the one obtained in 0.9 ± 0.2 mM, using a commercial spectrophotometric enzymatic kit.

## 1. Introduction

The inclusion of nanomaterials has marked a turning point in biosensor development. With remarkable achievements in nanotechnology and nanoscience, nanomaterials-based electrochemical signal amplifications have great potential in improving both the sensitivity and selectivity of electrochemical biosensors [1,2]. The high sensitivity and selectivity of nanomaterials-based biosensors have led to great advances in the development of new methodologies for the early detection and diagnosis of disease associated biomarkers. Currently, the new synthetic methods available, allow preparation of a wide range of nanomaterials with tunable size, shape, surface functional groups, and physicochemical features [3,4]. In particular, a wide range of carbon nanomaterials have been explored as potential base platforms for the development of biosensing systems [5]. Among them, carbon nanotubes and graphene are the most employed ones, due to their unique mechanical, electrical, thermal, and optical properties. However, these carbon nanomaterials have significant drawbacks, derived from the difficulty of fabrication and high cost for commercial production [6].

Electrochemical biosensors that combine enzymes and carbon nanomaterials, joining the recognition and catalytic properties of the enzyme with the electronic properties of the nanomaterial, lead to novel devices with synergistic properties that originate from the components of the hybrid nanocomposites. The potential biomedical applications of these nanomaterials-based electrochemical biosensors have been summarized in some recent review articles [7,8].

Recently, a new member of the carbon nanomaterial family, carbon nanodots (CNDs), have gained attention because of their fine properties, water solubility, low cytotoxicity, high luminescence, and good conductivity [9]. Moreover, these quasi-spherical particles, with sizes below 10 nm, are typically constituted of carbon, hydrogen, and oxygen atoms, and are known for their benign, abundant, and inexpensive nature [10]. Among different synthesis protocols for producing CNDs, the bottom-up approach has shown that specific heteroatoms can be introduced during the synthesis by selecting the starting material [11,12], which is very convenient for the purpose of endowing them with specific functionalities. Furthermore, depending on the precursors employed in their synthesis, CNDs are surrounded by different functional groups including, among others, hydroxyl, carboxyl, and amide groups, which facilitate the immobilization of biomolecules. Hence, due to their ability to be modified with a wide variety of biomolecules, and in conjunction with the excellent properties mentioned above, CNDs have already been employed in many biological applications [13]; as well as others, such as photocatalysis and solar cell development [14,15,16,17].

Concerning the employment of CNDs for electrochemical biosensors, it should be highlighted that despite the previously mentioned advantages, very few attempts to incorporate CNDs into electrodes are reported. Previous works reporting the application of CNDs in electrochemical sensors are focused on the electrocatalytic properties of this nanomaterial towards hydrogen peroxide [18] and oxygen reduction [19], exploited for glucose biosensing [20,21] and peroxide [18], dopamine [22], 2,4,6-trinitrotoluene [23], patulin [24], and DNA sensing [25].

In the present work, we synthesized carbon dots using a thermal carbonization method [26] using tris(hydroxymethyl)aminomethane (TRIS) and ethyleneglycol bis-(2-aminoethyl ether)-N,N,N′,N′-tetraacetic acid (EGTA). We have studied the formation of bioconjugates between this nanomaterial and several oxidoreductases as promising approaches for electrochemical biosensing applications. In particular, the effect of CNDs in the response of lactate oxidase, glucose oxidase, and uricase based biosensors was studied. In order to perform an exhaustive study of the approach, lactate oxidase was taken as a model, and a L-lactate biosensor was developed. L-lactate is an interesting biomarker [27] for different pathologies [28,29,30,31,32], an indicator or prediction of exercise performance and control of training [33,34], as well as a chemical indicator of food taste [35,36]. Hence, the reliable monitoring of L-lactate is essential not only for clinical diagnostics, but also for sports medicine, biotechnology, and food analysis. To our knowledge, this is the first reagent-less electrochemical L-lactate biosensor that includes CNDs as nanomaterials.

## 2. Materials and Methods

### 2.1. Reagents and Apparatus

l-(+)-lactic acid lithium salt 97%, glucose, uric acid, Nafion^®^, sulfuric acid, ethyleneglycol bis-(2-aminoethyl ether)-N,N,N′,N′-tetraacetic acid (EGTA), and tris(hydroxymethyl)aminomethane (TRIS) were purchased from Sigma-Aldrich (Merck). Other chemicals used in this work were of analytical grade quality and used without any further purification. Lactate oxidase (LOx, EC 1.1.3.2 from microorganism) lyophilized powder was obtained from Orion High Technologies S.L. Glucose oxidase (GOx, EC 1.1.3.4 from *Aspergillus niger*), uricase (UOx, EC 1.7.3.3 from *Candida* sp.), and ascorbate oxidase (EC 1.10.3.3 from *Cucurbita* sp.) lyophilized powders, as well as the human serum sample, were obtained from Sigma-Aldrich (Merck). The enzymatic kit for L-lactate quantification (K-LATE 07/14) was obtained from Megazyme (Ireland). Stock solutions were prepared in 0.1 M phosphate buffer (pH 7.0) for LOx (200 U/mL) and GOx (636 U/mL). In the case of UOx, the stock solution (50 U/mL) was prepared in 0.1 M borate buffer (pH 8.5). All stock solutions were stored at –20 °C. Purification of water was carried out in a Millipore Milli-Q system.

A double beam PharmaSpec UV-1700 series (Shimadzu Corporation) was used to record optical absorption spectra. In order to use a small sample amount, low volume 1.0 cm quartz cuvettes from Hellma Analytics were employed.

Emission spectra were obtained using a Cary Eclipse spectrofluorimeter from Varian.

Electrochemical measurements were performed with a potentiostat PGSTAT 302N from Metrohm Autolab. Four-millimetre diameter screen-printed gold electrodes (SPAuE, Metrohm DropSens), including a silver pseudoreference electrode and a gold counter electrode, were used. For Electrochemical Impedance Spectroscopy (EIS) experiments, an equimolar (10 mM) K_3_Fe(CN)_6_/K_4_Fe(CN)_6_ mixture in a 0.1 M phosphate buffer solution (pH 7.0) was used. Impedance measurements were recorded in the 10^5^–10^−2^ Hz frequency range, with a sinusoidal potential modulation of ±10 mV in amplitude.

An inverted microscope Axiovert200 (Zeiss), coupled to a Charge-Coupled Device (CCD) monochrome camera, was employed to register the fluorescence images. A SPECTRA-X (LUMENCOR) was used as the illumination source with a 10X/0.45 Plan/Apochromat Ph 1. A DAPI (395/25) objective and DAPI (432/36) excitation and emission filters, respectively, were used.

Atomic force microscopy (AFM) studies were performed with an Agilent 5500 microscope with an Olympus cantilever (RC800PSA, 200_20 mm). Tapping-mode in liquid medium has been used to image the enzyme LOx deposits on gold substrates.

### 2.2. Procedures

#### 2.2.1. Synthesis of Carbon Nanodots

CNDs were synthesized from ethyleneglycol bis-(2-aminoethyl ether)-N,N,N′,N′-tetraacetic acid (EGTA) and tris(hydroxymethyl)aminomethane (TRIS), as described by Ahmed et al [26]. For that purpose, EGTA (0.8 g) and TRIS (1.0 g) were dissolved in purified water (30 mL). The solution was then heated up to 150 °C to form a yellow gel, and washed with five portions of water (1 mL). After that, the temperature was raised to 180 °C, and the gel became dark orange. It was then diluted in water (25 mL) and passed through a nylon filter (0.45 µm). The final purification was carried out by dialyzing the solution (MWCO, 3.5 KDa) for three days. It was then kept at 4 °C until use. From the elemental analysis, a concentration of 3.5 µM for the stock solution was estimated [25].

#### 2.2.2. Bioconjugate Enzyme-CNDs (Enz–CND) Preparation

Bioconjugates of LOx, GOx, or UOx, and CNDs, were prepared by mixing 25 µL of the stock solution of CNDs and 25 µL of the stock solution of the corresponding enzyme, allowing them to interact for one hour. Subsequently, to remove the excess of reagents, the bioconjugate was purified using an Amicon Ultra Centrifugal Filter (Millipore) by centrifugation at 9520 RCF (10,000 rpm) for 10 min at 4 °C.

#### 2.2.3. Fluorescence Microscopy and AFM Samples Preparation

Samples for fluorescence microscopy were prepared by drop casting 50 µL of the bioconjugate LOx–CNDs onto a glass slide (fluorescence microscopy) or gold substrates (AFM) and allowing it to dry for 24 h.

#### 2.2.4. Biosensor Preparation

For the first step, the SPAuE was activated in 0.1 M H_2_SO_4_ by cycling the potential (10 scans) from –0.2 and 1.2 V at 0.10 V/s. Then, 10 µL of the LOx–CNDs, GOx–CNDs, or UOx–CNDs bioconjugate solution was deposited onto the activated gold electrode surface and air dried (Enz–CNDs/SPAuE).

#### 2.2.5. Determination of L-lactate in Human Serum

The developed biosensor was employed in the quantification of L-lactate in human serum. Deproteinization was needed as a sample pretreatment. For that reason, equal volumes (1.0 mL) of serum and cold 1 M perchloric acid were mixed. The solution was agitated and centrifuged at 1720 RCF (4250 rpm) for 10 min. The supernatant was kept and neutralized with 1 M NaOH. After that, 0.3 mL of the neutralized sample was diluted in 3.0 mL of 0.1 M phosphate buffer (pH 7.0). The results obtained using the standard addition method were validated towards a commercial enzymatic assay kit.

## 3. Results and Discussion

Electrochemical biosensors that combine enzymes and nanomaterials integrate the recognition and catalytic properties of enzymes with the electronic properties of various nanomaterials. They provide novel constructs with synergistic properties that originate from the components of the hybrid nanocomposites. Among carbon-based nanomaterials, CNDs have attracted great interest, because they can be easily produced from a wide range of raw materials, and excel with their robust chemical inertness, high solubility in aqueous media, and biocompatibility.

The CNDs employed in this work were prepared using a previously described method, by carbonization of EGTA and TRIS, and characterized using different techniques [26]. The resulting nanoparticles are monodispersed and have an average size of 3.4 nm, ranging from 2 to 5 nm in diameter, as demonstrated by dynamic light scattering (DLS). The FTIR spectrum indicates that these CNDs have amide and alcohol groups on their surface. This interesting surface chemistry makes it possible to conjugate them with biomolecules to form nanohybrids. To assess whether this is the case, in this work, the formation of bioconjugates between CNDs and three different oxidases (lactate oxidase (LOx), glucose oxidase (GOx), and uricase (UOx)) was reported. Both components were mixed and allowed to react for 15 to 90 minutes, as described in the experimental section. At neutral pH, enzymes (isoelectric point 4.0–5.0) are negatively charged. Thus, they can undergo an electrostatic interaction, with positively charged functional groups present at the CNDs surface, or they can be adsorbed on the carbon-based nanomaterial via π–π stacking interactions and hydrogen bonding. Moreover, we have proved the utility of the resulting bioconjugates for biosensor development. The biosensor was developed by direct modification of a screen-printed gold electrode with the bioconjugate, as described in the experimental section.

**Figure 1:**
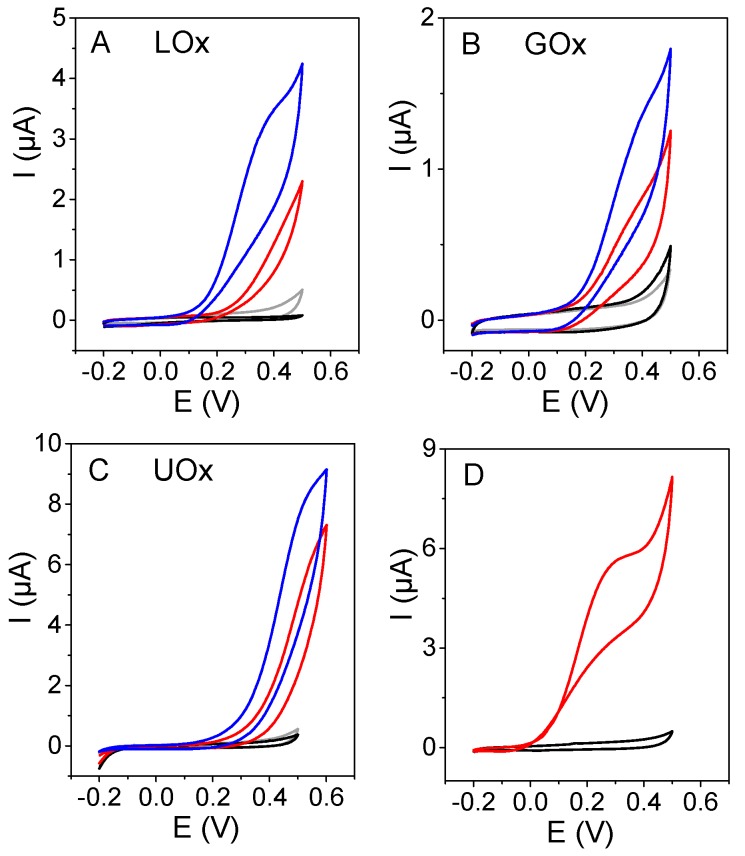
Cyclic voltammograms of SPAuE (black), Enz/SPAuE (red), Enz–CNDs/SPAuE (blue) when (**A**) LOx, (**B**) GOx, and (**C**) UOx were used, in the presence of 0.5 mM L-lactate, 1.0 mM glucose, and 1.0 mM uric acid (A, B, and C, respectively) and Enz–CNDs/SPAuE in the absence of substrate (grey). (**D**) Cyclic voltammograms of CNDs/SPAuE in 0.1 M phosphate buffer (pH 7.0) in the absence (black) and presence (red) of 0.5 mM H_2_O_2_. Scan rate: 0.01 V/s.

Figure 1 shows the response of three different biosensors prepared with each bioconjugate (Lox–CNDs, Gox–CNDs, or UOx–CNDs), in the absence and in the presence of L-lactate, glucose, and uric acid, respectively. As a comparison, the response of biosensors prepared by adsorption of only the enzyme (LOx, GOx, or UOx) on the electrode surface to L-lactate, glucose, and uric acid, are also included.

**Scheme 1:**
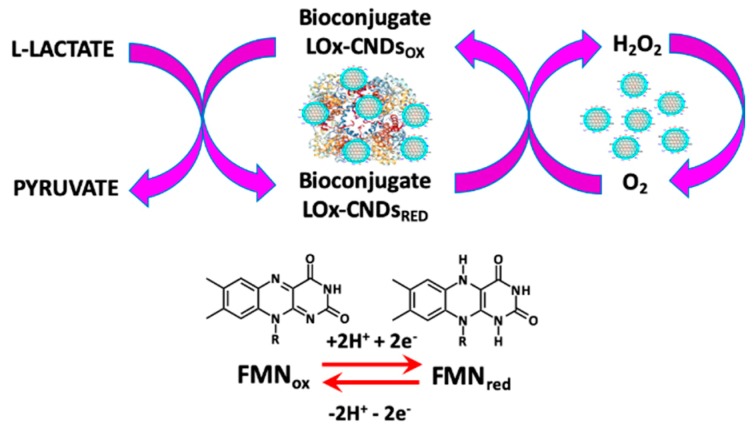
Enzymatic process for the bioconjugate LOx–CNDs.

Oxidoreductases catalyze the oxidation of the corresponding substrate (L-lactate, glucose, uric acid) and the product (pyruvate, gluconic acid, allantoin). These enzymes contain, in their active center, the oxidized form of a flavin nucleotide capable of oxidizing the substrate to give the reaction product. The regeneration of the active form of the cofactor in the active center takes place with the actions of the molecular oxygen, which is reduced to hydrogen peroxide. The enzymatically generated hydrogen peroxide is oxidized on the electrode surface, a process that is catalyzed by the CNDs present in the bioconjugate, as shown in Scheme 1 for the bioconjugate LOx–CNDs and the corresponding substrate L-lactate.

The biosensor response is based on the detection of the electrochemically active hydrogen peroxide generated in the process, which is proportional to the substrate concentration present in the solution.

As can be observed in Figure 1, in all cases, the bioconjugate provides higher peak currents at a lower potential, compared to those obtained when the biosensor was prepared with the enzyme alone. CNDs act as a promoter of the electrooxidation of peroxide and have a synergetic effect in the enzymatic activity, particularly in the case of the LOx–CNDs.

In order to assess if the measured current comes from the enzymatically generated hydrogen peroxide, the response of a CNDs modified electrode to H_2_O_2_ was also studied. A well-defined peak current at +0.30 V was observed (Figure 1D), confirming that the biosensor response was due to the oxidation of the hydrogen peroxide generated in the enzymatic reaction.

As mentioned above, the best results were obtained for the LOx–CNDs/L-lactate system. Moreover, L-lactate has an important role as biomarker. Hence, in the following work as a model system, we developed a CNDs-based biosensing platform employing LOx. This prototype was employed to perform an exhaustive study of the system to confirm the potential utility of this nanomaterial in the construction of improved biosensing devices.

### 3.1. LOx–CNDs Bioconjugate Characterization

**Figure 2:**
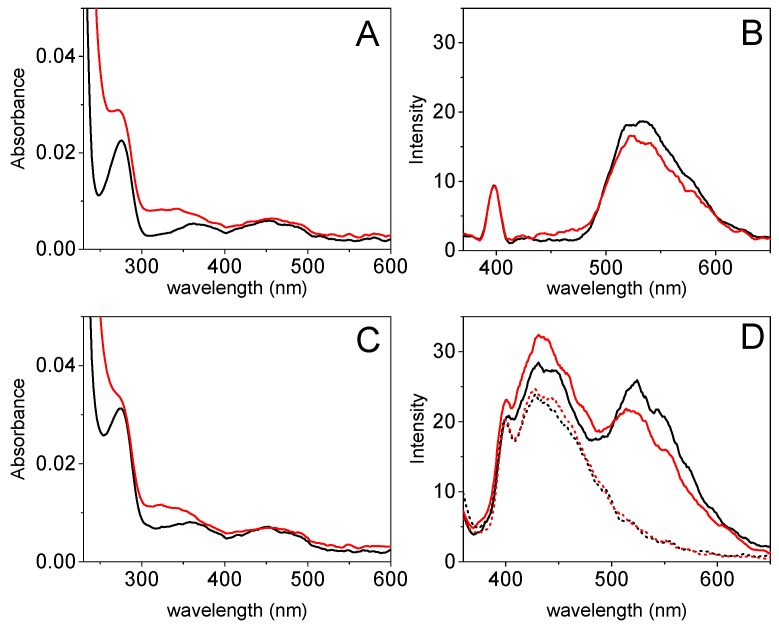
UV-Visible absorption spectra of (**A**) LOx and (**C**) the bioconjugate LOx–CNDs, and fluorescence emission spectra (λ_ex_= 350 nm) of (**B**) LOx and (**D**) the bioconjugate LOx–CNDs (solid) and CNDs (dotted), in the absence (black) and in the presence (red) of 0.5 mM L-lactate in 0.1 M phosphate buffer (pH 7.0). Taken after 30 minutes L-lactate addition.

The features of the LOx–CNDs bioconjugate were studied using UV-visible absorption and fluorescence spectroscopy, and compared with those of LOx. The spectrophotometric behavior of the enzyme (Figure 2A black line) and the bioconjugate (Figure 2C black line) show an absorption band at 275 nm, ascribed to the apoenzyme [37]; as well as two less defined bands at 370 and 455 nm, which corresponds to the oxidized form of the Flavin mononucleotide (FMN) cofactor [38]. The fluorescence spectrum of the enzyme (see Figure 2B black line) shows two bands at 398 and 530 nm, exciting at 350 nm. The band at 530 nm is related to FMN [39], and the one at 398 nm corresponds to the water Raman peak.

Upon addition of L-lactate (red lines), the absorption bands at 275 and at 370 nm of both LOx (Figure 2A) and the bioconjugate (Figure 2C) increase, due to the absorption of the pyruvate generated in the enzymatic reaction [40]. These results confirm that the bioconjugate behaves like the enzyme. The emission band at 398 nm in the LOx fluorescence spectrum (see Figure 2B red line) remains unmodified. However, the band at 530 nm shows a decrease of about 12% as a consequence of the quenching effect of the hydrogen peroxide generated in the enzymatic reaction. The absorbance and emission spectra of L-lactate alone, recorded in the same conditions, show no bands (data not shown).

The fluorescence spectrum of the LOx–CNDs bioconjugate (Figure 2D black line), like in the case of the absorbance spectrum, shows the enzyme band at 530 nm besides the characteristic band at 450 nm of the CNDs. As it can be seen, the band ascribed to the enzyme shows a lower fluorescence in the presence of L-lactate (Figure 2D red line), whereas the band for the CNDs of the bioconjugate increases its fluorescence. This fact is due to the interaction of the pyruvate generated in the enzymatic reaction with CNDs. In contrast, there are no changes in the fluorescence band of the CNDs upon the addition of L-lactate (Figure 2D red dotted line), confirming the absence of reaction between the enzymatic substrate and the nanomaterial by itself.

The above described results point to the formation of a bioconjugate between LOx and CNDs. It seems to involve interactions that do not imply changes, neither in the electronic structure of the enzyme, nor in its activity towards L-lactate.

**Figure 3:**
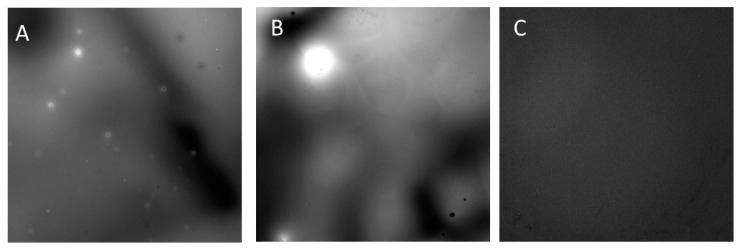
Fluorescence micrographs of (**A**) CNDs, (**B**) LOx–CNDs bioconjugate, and (**C**) LOx, at 20× magnification.

In order to assess the LOx–CNDs bioconjugate formation, and based on the fluorescence property of the CNDs, a morphological study of the bioconjugate using fluorescence microscopy was also carried out. Figure 3 shows the fluorescence micrographs (obtained via excitation from 375 to 420 nm, and emission from 396 to 468 nm) of CNDs (A), LOx–CNDs bioconjugate (B), and LOx (C) samples. As can be observed, the LOx–CNDs bioconjugate (B) shows fluorescence which comes from CNDs, since LOx by itself does not present fluorescence under the experimental conditions employed (see control image (C)). On the contrary, CNDs aggregates possess a strong fluorescence (see micrograph A). The bioconjugate displays bigger fluorescence spots than that observed for the CNDs alone. This is probably due to the formation of larger CNDs aggregates due to the presence of the enzyme. These images suggest that the enzyme acts as a binder, promoting the formation of big aggregates between enzymes and CNDs. These results indicate that CNDs interact with LOx, and confirm again, the formation of the bioconjugate of CNDs with LOx_._

### 3.2. LOx–CNDs Based Biosensor

Several parameters have been optimized for biosensor development. Among them, we have studied the influence of the number of CNDs employed, on the formation of the bioconjugate and reaction time. For that purpose, LOx–CNDs bioconjugates were prepared by mixing different volumes of the CNDs stock solution with 1 U of LOx for 15, 30, 60, and 90 min. The best result was obtained using 5 µL of the CNDs stock solution, and 60 minutes (data not shown).

Once the biosensor was developed, the first step was the characterization of the device by using different techniques.

**Figure 4:**
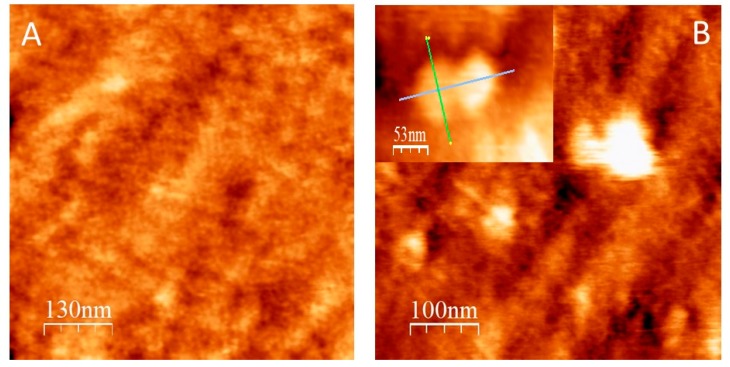
Tapping-mode AFM topographic images of a (**A**) LOx–CNDs bioconjugate, and (**B**) LOx. Inset in B shows a photograph of a dimer formed by the LOX. All the experiments were carried out on a gold plate.

The morphology of the electrode surface modified with the bioconjugate LOx–CNDs was studied with AFM, and compared to that of an electrode modified with LOx alone. The topographic image of a LOx–CNDs sample (Figure 4A) shows evidence of LOx binding to the surface. It can be observed that the surface is decorated with a film composed of globules distributed along it. The expected size of the individual proteins is 50 × 100 × 100 Å [41]. The globular disposition of the individual LOx molecules over the gold surface is observed, with a mean height of approximately 10 nm, which agrees with the expected size (Figure 4B). The absence of holes randomly distributed in the sample, indicates that there is no sublayer of LOX molecules or CNDs. This suggests that it results in a complete monolayer; whereas in the LOx sample, the presence of holes in the surface is clear, as can be seen in Figure 4B.

The complete topography of LOx cannot be defined with the AFM tip in the LOx–CNDs sample, because the enzyme molecules are linked together. This may be due to the fact that the presence of the CNDs favors the generation of a compact and ordered monolayer of proteins, with a very homogenous distribution that forms a tetragonal network.

**Figure 5:**
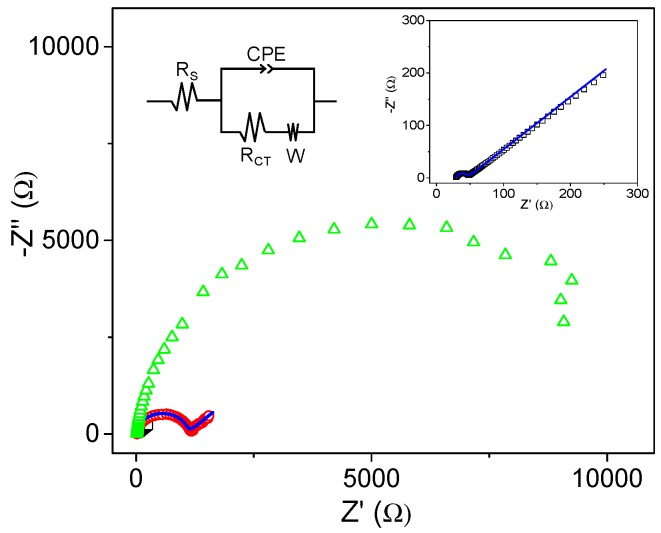
Nyquist diagrams in 0.1 M phosphate buffer (pH 7.0) containing 10 mM K_3_Fe(CN)_6_/10 mM K_4_Fe(CN)_6_ for SPAuE (□), LOx/SPAuE (∆) and LOx–CNDs/SPAuE (○). Blue lines correspond to the fitting of the experimental data, to the shown equivalent circuit.

Electrochemical impedance analysis (EIS) is a powerful tool for studying the interfacial properties of modified electrodes. Therefore, it was performed to monitor changes in the properties of the electrode surface after modification with the bioconjugate. As a comparison, the behavior of electrodes modified with LOx alone was also studied with the same technique. Figure 5 shows the electrochemical impedance spectra, using [Fe(CN)_6_]^4−/3−^ as a redox probe of SPAuE, LOx/SPAuE, and LOx–CNDs/SPAuE. SPAuE and LOx–CNDs/SPAuE diagrams were fitted according to the equivalent circuit shown in Figure 5. The impedance spectrum of SPAuE (Figure 5 black) shows a charge-transfer limited process at higher frequencies (semicircle), with a charge-transfer resistance (R_CT_) value of 20 Ω and a diffusion-limited process at lower frequencies (straight line portion). When LOx was immobilized on the electrode surface, a big arc appeared (Figure 5 green). It does not form a full semicircle, since at low frequencies data are randomly distributed. This result agrees well with an increase in the charge-transfer resistance on the electrode surface, due to the fact that the enzymatic layer forms an insulating barrier that blocks the redox probe diffusion from the bulk solution to the electrode surface. Moreover, the increase in the semicircle diameter proved the hindrance of the electron flow, confirming the well-known poor electrical properties at low frequencies of most biomolecules [42,43]. However, when the electrode was modified with the LOx–CND bioconjugate, the Nyquist diagram shows again a typical pattern of a simple redox process under kinetic and diffusion control. In this case, the obtained R_CT_ value of 1.1 kΩ indicates facilitated charge-transfer limited processes, and confirms the increase of the rate of electron transfer due to the presence of the CNDs in the bioconjugate.

### 3.3. Biosensor Response

**Figure 6:**
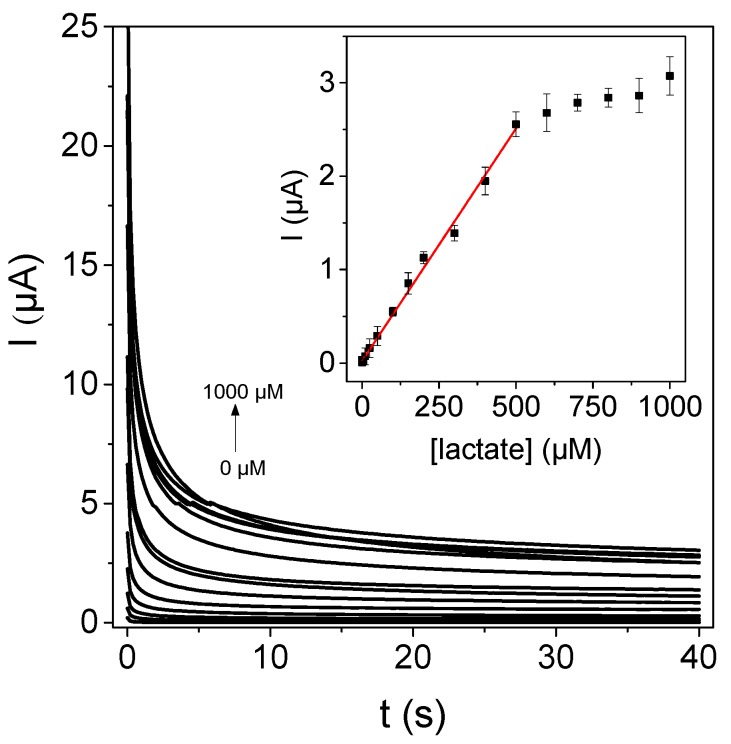
Chronoamperometric biosensor response constructed from the bioconjugate LOx–CNDs in the presence of increasing L-lactate concentrations in 0.1 M phosphate buffer (pH 7.0). Inset: calibration curve.

Once the different steps for the biosensor development were characterized and the conditions were optimized, the biosensor chronoamperometric response at +0.4 V to the increasing L-lactate concentrations were recorded (see Figure 6), and the analytical parameters were evaluated. The steady state is reached after 38 s. The response fits well to a Michaelis–Menten curve, indicating that the enzymatic reaction is responsible for the biosensor response. The plot shows a linear behavior from 3.0 µM up to 500 µM; and from the slope, a sensitivity of 4.98 × 10^−3^ µA·µM^−1^ was calculated. Detection and quantification limits of 0.9 and 3.0 µM, respectively, were estimated from the standard deviation of the background current. The RSD value of the response of three different biosensors prepared in the same manner, were employed to evaluate the reproducibility of the biosensor. It was found to be 8.6%. These properties compare well with other recently published L-lactate electrochemical biosensors based on LOx, summarized in Table 1 [44,45,46,47,48,49,50], with the advantage of this being simpler and prepared with low cost materials.

### 3.4. Interfering Substances Study

Selectivity is an important parameter in the performance of a biosensor. Therefore, with the aim of evaluating the applicability of the developed biosensor for lactate quantification in a sample such as human serum, the effect of the potential interfering compounds more commonly found in this sample was studied. Thus, the biosensor response to L-lactate, in the presence of two different concentrations of glucose, ascorbic acid, uric acid, and acetaminophen, was recorded. As can be seen in Table 2, the response is not significantly affected by the presence of glucose or uric acid. However, both acetaminophen and ascorbic acid have an effect on the biosensor response, especially when they are in the same concentration as the analyte. In the case of ascorbic acid, this interference is caused by the fact that ascorbic acid is a reducing agent, and the measured compound, the enzymatically generated peroxide, is an oxidant; therefore, a reaction between them occurs. With the aim of improving the biosensor performance and minimizing the effect of ascorbic acid, the electrode surface was covered by a Nafion® layer before preparing the biosensor, in order to impede the ascorbate ion from reaching the negatively charged electrode surface. This strategy reduces the interference caused by ascorbic acid by about 20% when compared with the value obtained without Nafion^®^ (see Table 2), but it still causes a negative interference in the determination of L-lactate. For that reason, a second approach, including ascorbate oxidase (AOx; 10 U) into the LOx–CNDs bioconjugate in biosensor development, was tried. Ascorbate oxidase catalyzes the oxidation of L-ascorbate according to reaction (1), reducing the concentration of ascorbic acid present in the solution.

2 L-ascorbate + O_2_ → 2 Dehydroxyascorbate + H_2_O (1)

In this case, the analytical signal of the biosensor (see Table 2) is almost the same as the one obtained in the absence of ascorbic acid. This approach proves to be a good strategy to eliminate the ascorbic acid effect in the biosensor response to L-lactate. Strategies to eliminate the ascorbic acid interference.

### 3.5. Determination of L-lactate in Human Serum

L-lactate is produced in the human body with the anaerobic metabolism of glucose. Normal levels in blood vary between 0.5 and 1.5 mM [34]. An increase in this concentration, besides as an exercise indicator, may be a biomarker for different pathologies [28,29,30,31,32]. Therefore, L-lactate determination is important in clinical and sports areas. For that reason, the biosensor performance in the L-lactate quantification in a commercial human serum sample was studied. After deproteinization, 0.3 mL of the sample was diluted in 3.0 mL of 0.1 M phosphate buffer (pH 7.0). Due to the sample complexity, and with the aim of minimizing matrix effects, the standard addition method was employed. A L-lactate concentration in the serum extract of 0.9 ± 0.3 mM (n = 3) was calculated. In order to check the accuracy of this result, it was compared with the one obtained using a commercial spectrophotometric enzymatic kit. In this case, a value of 0.9 ± 0.2 mM was obtained, which matches the one obtained using the biosensor, and confirms that the developed biosensor can be employed for L-lactate determination in serum samples in a fast and inexpensive way.

## 4. Conclusions

We demonstrated the suitability of employing carbon nanodots (CNDs) as a low-cost nanomaterial for developing improved electrochemical enzyme-based biosensors. Lactate oxidase was chosen as a model system. CNDs were conjugated to the enzyme by simply mixing both components, due to the CNDs interesting surface chemistry. The resulting enzyme–CNDs bioconjugate shows, in addition to the enzymatic activity, an electrocatalytic activity towards the oxidation of peroxide. It allows detection in this product of the enzymatic reaction at a low potential of +0.35 V. Hence, when the bioconjugate is deposited onto screen-printed gold electrodes, it results in an efficient disposable biosensor for L-lactate determination with enhanced performance. It shows a wide linear range and a low detection limit, when used to detect this analyte in human serum. The developed biosensor shows several advantages, compared to others developed employing nanomaterials, such as an easy fabrication procedure using low-cost materials.

## Figures and Tables

**Table 1:** Analytical parameters of some L-lactate biosensors based on LOx.

Electrode	Modification	LOD (μM)	Linear Range (μM)	Matrix	Reference
SPCE	LOx/PtNp–CNF–PDDA	11.1	25–1500	sweat	[44]
GCE	LOx/FcMe_2_–LPEI	3	up to 5000	-	[45]
Au	LOx–BSA–GA	5	5–1000	wine	[46]
SPCE	LOx/rGO–DHS	2.9	up to 800	white wine	[47]
SPCE	LOx–Cu–MOF/CS/Pt	0.75	0.75–1000	wine, saliva, sweat	[48]
SPCE	LOx–[Fe(CN)_6_]^3+^	-	390–16600	tears	[49]
Au	LOx/pMB/Au–MWCNTs	2.4	10–200	interstitial fluid	[50]
SPAuE	LOx–CNDs	0.9	up to 500	human serum	this work

SPCE: screen printed carbon electrode; PtNp–CNF: platinum nanoparticle decorated carbon nanofibers; PDDA: poly(diallyldimethylammonium) chloride; GCE: glassy carbon electrode; FcMe2: dimethylferrocene; LPEI: linear polyethyleneimine; BSA: bovine serum albumin; GA: glutaraldehyde; rGO-DHS: 3,4-dihydroxysalophen modified reduced graphene oxide; Cu-MOF: copper metallic framework; CS: chitosan; pMB: poly-methylene blue; MWCNTs: multiwalled carbon nanotubes.

**Table 2:** Effect of the potentially interfering compounds on the biosensor response.

Substance	Relative intensity	
	1:0.1	1:1
Glucose	1.1	1.1
Uric acid	1.0	1.1
Acetaminophen	1.3	2.3
Ascorbic acid	0.5	0.4
Strategies to eliminate the ascorbic acid interference		
Ascorbic acid		
Nafion^®^	0.7	0.6
AOx	0.9	1.1

Relative intensity = IL + I/IL. IL: biosensor response to 0.50 mM·L-lactate; IL + I: biosensor response to 0.50 mM·L-lactate in the presence of interfering substance at a final concentration of 0.05 mM (1:0.1) or 0.5 mM (1:1).

## References

[1] Asadian E., Ghalkhani M., Shahrokhian S. (2019). Electrochemical Sensing Based on Carbon Nanoparticles: A Review. Sens. Actuators B Chem..

[2] Manikandan V.S., Adhikari B., Chen A. (2018). Nanomaterial Based Electrochemical Sensors for the Safety and Quality Control of Food and Beverages. Analyst.

[3] Ansari S.M., Sinha B.B., Phase D., Sen D., Sastry P.U., Kolekar Y.D., Ramana C.V. (2019). Particle Size, Morphology, and Chemical Composition Controlled CoFe2O4 Nanoparticles with Tunable Magnetic Properties Via Oleic Acid Based Solvothermal Synthesis for Application in Electronic Devices. ACS Appl. Nano Mater..

[4] Chen M., He Y., Liu X., Zhu J., Liu R. (2017). Synthesis and Optical Properties of Size-Controlled Gold Nanoparticles. Powder Technol..

[5] Wang Z., Dai Z. (2015). Carbon Nanomaterial-Based Electrochemical Biosensors: An Overview. Nanoscale.

[6] Borenstein A., Hanna O., Attias R., Luski S., Brousse T., Aurbach D. (2017). Carbon-Based Composite Materials for Supercapacitor Electrodes: A Review. J. Mater. Chem. A.

[7] Teradal N.L., Jelinek R. (2017). Carbon Nanomaterials in Biological Studies and Biomedicine. Adv. Healthc. Mater..

[8] Chen A., Chatterjee S. (2013). Nanomaterials Based Electrochemical Sensors for Biomedical Applications. Chem. Soc. Rev..

[9] Baker S.N., Baker G.A. (2010). Luminescent Carbon Nanodots: Emergent Nanolights. Angew. Chem. Int. Ed..

[10] Lim S.Y., Shen W., Gao Z. (2015). Carbon Quantum Dots and their Applications. Chem. Soc. Rev..

[11] Park Y., Yoo J., Lim B., Kwon W., Rhee S.W. (2016). Improving the Functionality of Carbon Nanodots: Doping and Surface Functionalization. J. Mater. Chem..

[12] Arcudi F., Dordevic L., Prato M. (2016). Synthesis, Separation, and Characterization of Small and Highly Fluorescent Nitrogen-Doped Carbon NanoDots. Angew. Chem. Int. Ed..

[13] Wang J., Qiu J. (2016). A Review of Carbon Dots in Biological Applications. J. Mater. Sci..

[14] Hong G., Diao S., Antaris A.L., Dai H. (2015). Carbon Nanomaterials for Biological Imaging and Nanomedicinal Therapy. Chem. Rev..

[15] Zhao A., Chen Z., Zhao C., Gao N., Ren J., Qu X. (2015). Recent Advances in Bioapplications of C-Dots. Carbon.

[16] Margraf J.T., Lodermeyer F., Strauss V., Haines P., Walter J., Peukert W., Costa R.D., Clark T., Guldi D.M. (2016). Using Carbon Nanodots as Inexpensive and Environmentally Friendly Sensitizers in Mesoscopic Solar Cells. Nanoscale Horiz..

[17] Hutton G.A.M., Martindale B.C.M., Reisner E. (2017). Carbon Dots as Photosensitisers for Solar-Driven Catalysis. Chem. Soc. Rev..

[18] Wang Y., Wang Z., Rui Y., Li M. (2015). Horseradish Peroxidase Immobilization on Carbon Nanodots/CoFe Layered Double Hydroxides: Direct Electrochemistry and Hydrogen Peroxide Sensing. Biosens. Bioelectron..

[19] Martinez-Perinan E., Bravo I., Rowley-Neale S.J., Lorenzo E., Banks C.E. (2018). Carbon Nanodots as Electrocatalysts Towards the Oxygen Reduction Reaction. Electroanalysis.

[20] Li H., Chen L., Wu H., He H., Jin Y. (2014). Ionic Liquid-Functionalized Fluorescent Carbon Nanodots and their Applications in Electrocatalysis, Biosensing, and Cell Imaging. Langmuir.

[21] Ji H., Zhou F., Gu J., Shu C., Xi K., Jia X. (2016). Nitrogen-Doped Carbon Dots as A New Substrate for Sensitive Glucose Determination. Sensors.

[22] Huang Q., Hu S., Zhang H., Chen J., He Y., Li F., Weng W., Ni J., Bao X., Lin Y. (2013). Carbon Dots and Chitosan Composite Film Based Biosensor for the Sensitive and Selective Determination of Dopamine. Analyst.

[23] Zhang L., Han Y., Zhu J., Zhai Y., Dong S. (2015). Simple and Sensitive Fluorescent and Electrochemical Trinitrotoluene Sensors Based on Aqueous Carbon Dots. Anal. Chem..

[24] Guo W., Pi F., Zhang H., Sun J., Zhang Y., Sun X. (2017). A Novel Molecularly Imprinted Electrochemical Sensor Modified with Carbon Dots, Chitosan, Gold Nanoparticles for the Determination of Patulin. Biosens. Bioelectron..

[25] Garcia-Mendiola T., Bravo I., Maria Lopez-Moreno J., Pariente F., Wannemacher R., Weber K., Popp J., Lorenzo E. (2018). Carbon Nanodots Based Biosensors for Gene Mutation Detection. Sens. Actuators B Chem..

[26] Ahmed G.H.G., Badia Laino R., Garcia Calzon J.A., Diaz Garcia M.E. (2015). Highly Fluorescent Carbon Dots as Nanoprobes for Sensitive and Selective Determination of 4-Nitrophenol in Surface Waters. Microchim. Acta.

[27] Freund Y., Delerme S., Goulet H., Bernard M., Riou B., Hausfater P. (2012). Serum Lactate and Procalcitonin Measurements in Emergency Room for the Diagnosis and Risk-Stratification of Patients with Suspected Infection. Biomarkers.

[28] Valenza F., Aletti G., Fossali T., Chevallard G., Sacconi F., Irace M., Gattinoni L. (2005). Lactate as a Marker of Energy Failure in Critically Ill Patients: Hypothesis. Crit. Care.

[29] Karlsson J., Willerson J.T., Leshin S.J., Mullins C.B., Mitchell J.H. (1975). Skeletal-Muscle Metabolites in Patients with Cardiogenic-Shock or Severe Congestive Heart-Failure. Scand. J. Clin. Lab. Investig..

[30] Sayeed M.M., Murthy P. (1981). Adenine-Nucleotide and Lactate Metabolism in the Lung in Endotoxin-Shock. Circ. Shock.

[31] DeBacker D., Creteur J., Zhang H.B., Norrenberg M., Vincent J.L. (1997). Lactate Production by the Lungs in Acute Lung Injury. Am. J. Respir. Crit. Care Med..

[32] Kruse J.A., Zaidi S., Carlson R.W. (1987). Significance of Blood Lactate Levels in Critically Ill Patients with Liver-Disease. Am. J. Med..

[33] Billat L.V. (1996). Use of Blood Lactate Measurements for Prediction of Exercise Performance and for Control of Training. Recommendations for Long-Distance Running. Sports Med..

[34] Stanley W.C., Gertz E.W., Wisneski J.A., Morris D.L., Neese R.A., Brooks G.A. (1985). Systemic Lactate Kinetics during Graded-Exercise in Man. Am. J. Physiol..

[35] Thierry A., Pogačić T., Weber M., Lortal S., Mozzi F., Raya R.R., Vignolo G.M. (2015). Production of Flavor Compounds by Lactic Acid Bacteria in Fermented Foods. Biotechnology of Lactic Acid Bacteria.

[36] Hemmi A., Yagiuda K., Funazaki N., Ito S., Asano Y., Imato T., Hayashi K., Karube I. (1995). Development of a Chemiluminescence Detector with Photodiode Detection for Flow-Injection Analysis and its Application to L-Lactate Analysis. Anal. Chim. Acta.

[37] Choong Y.S., Shepherd M.G., Sullivan P.A. (1975). Preparation of the Lactate Oxidase Apoenzyme and Studies on the Binding of Flavin Mononucleotide to the Apoenzyme. Biochem. J..

[38] Macheroux P. (1999). UV-Visible Spectroscopy as a Tool to Study Flavoproteins. Methods Mol. Biol..

[39] Lakowicz J.R. (2006). Principles of Fluorescence Spectroscopy.

[40] Griffith E.C., Carpenter B.K., Shoemaker R.K., Vaida V. (2013). Photochemistry of Aqueous Pyruvic Acid. Proc. Natl. Acad. Sci. USA.

[41] Leiros I., Wang E., Rasmussen T., Oksanen E., Repo H., Petersen S.B., Heikinheimo P., Hough E. (2006). The 2.1 Å Structure of Aerococcus Viridans L-Lactate Oxidase (LOX). Acta Crystallogr. Sect. F Struct. Biol. Commun..

[42] Kesik M., Ekiz Kanik F., Turan J., Kolb M., Timur S., Bahadir M., Toppare L. (2014). An Acetylcholinesterase Biosensor Based on a Conducting Polymer using Multiwalled Carbon Nanotubes for Amperometric Detection of Organophosphorous Pesticides. Sens. Actuators B Chem..

[43] Salazar P., Martín M., González-Mora J.L. (2019). In Situ Electrodeposition of Cholesterol Oxidase-Modified Polydopamine Thin Film on Nanostructured Screen Printed Electrodes for Free Cholesterol Determination. J. Electroanal. Chem..

[44] Lamas-Ardisana P.J., Loaiza O.A., Anorga L., Jubete E., Borghei M., Ruiz V., Ochoteco E., Cabanero G., Grande H.J. (2014). Disposable Amperometric Biosensor Based on Lactate Oxidase Immobilised on Platinum Nanoparticle-Decorated Carbon Nanofiber and Poly(Diallyldimethylammonium Chloride) Films. Biosens. Bioelectron..

[45] Hickey D.P., Reid R.C., Milton R.D., Minteer S.D. (2016). A Self-Powered Amperometric Lactate Biosensor Based on Lactate Oxidase Immobilized in Dimethylferrocene-Modified LPEI. Biosens. Bioelectron..

[46] Shkotova L.V., Piechniakova N.Y., Kukla O.L., Dzyadevych S.V. (2016). Thin-Film Amperometric Multibiosensor for Simultaneous Determination of Lactate and Glucose in Wine. Food Chem..

[47] Bravo I., Revenga-Parra M., Weber K., Popp J., Pariente F., Lorenzo E. (2018). One-Step Reduced/Quinone Functionalized Graphene Oxide as Reagentless Lactate Biosensing Platform. Sens. Actuators B Chem..

[48] Cunha-Silva H., Arcos-Martinez M.J. (2018). Dual Range Lactate Oxidase-Based Screen Printed Amperometric Biosensor for Analysis of Lactate in Diversified Samples. Talanta.

[49] Lin C., Hiraka K., Matloff D., Johns J., Deng A., Sode K., La Belle J. (2018). Development Toward a Novel Integrated Tear Lactate Sensor using Schirmer Test Strip and Engineered Lactate Oxidase. Sens. Actuators B Chem..

[50] Bollella P., Sharma S., Cass A.E.G., Antiochia R. (2019). Microneedle-Based Biosensor for Minimally-Invasive Lactate Detection. Biosens. Bioelectron..

